# Postsurgical Thrombotic Microangiopathy after Thoracoabdominal Aortic Aneurysm Repair: A Case Report

**DOI:** 10.3400/avd.cr.26-00038

**Published:** 2026-07-07

**Authors:** Jien Saito, Shinji Kamiya, Hideki Sasaki

**Affiliations:** 1 Department of Cardiovascular Surgery, Nagoya City University East Medical Center, Nagoya, Aichi, Japan; 2 Department of Cardiovascular Surgery, Hokkaido University, Sapporo, Hokkaido, Japan

**Keywords:** thrombotic microangiopathy, aortic surgery, plasma exchange

## Abstract

Postsurgical thrombotic microangiopathy (TMA) is a highly lethal complication. We report a 71-year-old woman who developed TMA following open thoracoabdominal aortic aneurysm repair. From postoperative day 2, she presented with progressive thrombocytopenia, acute kidney injury, and hemolytic anemia. Therapeutic plasma exchange (PE) and hemodialysis were promptly initiated upon clinical suspicion, without waiting for ADAMTS13 results. In conclusion, we experienced a case of postsurgical TMA triggered by profound surgical stress, in which prompt initiation of PE was effective. The patient successfully recovered from the acute phase and has remained in remission for over 3 years.

## Introduction

Thrombotic microangiopathy (TMA) occurring after aortic treatment represents a life-threatening postoperative complication characterized by microangiopathic hemolytic anemia (MAHA), consumptive thrombocytopenia, and organ injury resulting from microthrombus formation, particularly acute kidney injury (AKI).^1)^ Recently, postsurgical TMA, triggered by the surgical procedure itself, has been increasingly recognized as a potential cause of unexplained severe AKI following cardiovascular surgery. Aortic surgery has been identified as a strong and independent risk factor for TMA (odds ratio 3.26),^2)^ with a reported incidence of 4.5%–5%.^3)^

However, TMA after cardiovascular surgery is rare, and its differential diagnosis is challenging because postoperative thrombocytopenia and severe AKI are also common in complications such as infection, disseminated intravascular coagulation (DIC), and heparin-induced thrombocytopenia (HIT).^4)^ Although the International Society on Thrombosis and Haemostasis (ISTH) guidelines recommend prompt measurement of ADAMTS13 activity,^5)^ this condition may be overlooked in routine postoperative care.

We report a case of severe postoperative TMA complicated by AKI and spinal cord infarction (SCI) in the early period following thoracoabdominal aortic graft replacement. TMA was suspected promptly, and while awaiting exclusion of thrombotic thrombocytopenic purpura (TTP) based on ADAMTS13 activity, immediate treatment with a tandem circuit of plasma exchange (PE) and hemodialysis (HD) was initiated. The patient survived the acute phase and has remained recurrence-free for 3 years without maintenance therapy with complement inhibitors.

## Case Report

The patient was a 71-year-old woman with no significant family history and no notable past medical history. In June 2021, she presented emergently with an impending rupture of a dissecting aortic aneurysm (maximum diameter 100 × 85 mm) and underwent Zone 2 single-vessel debranching thoracic endovascular aortic repair (TEVAR) combined with the Candy-Plug (**Fig. 1A**–**1C**). Later that year, she required local wound management for a cervical surgical site infection. She subsequently underwent bilateral bronchial artery coil embolization to address aneurysm enlargement and esophageal compression due to a Type II endoleak originating from the bronchial arteries. As the aneurysm continued to enlarge thereafter, thoracoabdominal aortic graft replacement was scheduled. Preoperative renal function (serum creatinine [SCr] 0.76 mg/dL; estimated glomerular filtration rate 57 mL/min/1.73 m^2^), platelet count (PLT 167000/μL), and other coagulation and fibrinolytic parameters were within normal limits.

**Fig. 1:**
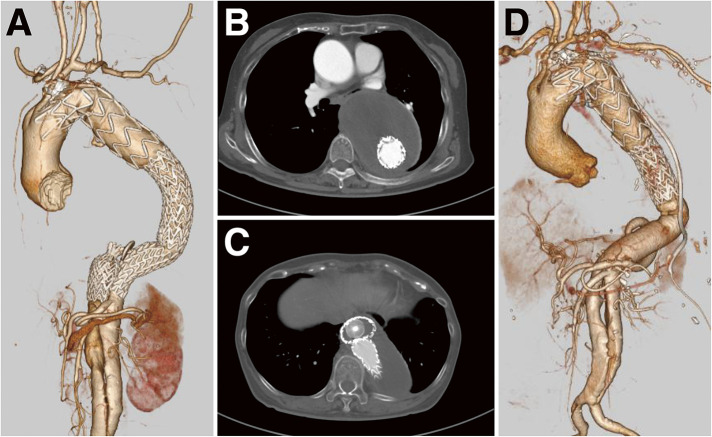
Pre- and postoperative contrast-enhanced CT images. (**A**) Preoperative 3D volume-rendered image showing an extensive thoracoabdominal aortic aneurysm with prior TEVAR. (**B**) Preoperative axial image at the level of the maximum aneurysm diameter (100 × 85 mm). (**C**) Preoperative axial image at the level of the prior Candy-Plug. (**D**) Postoperative 3D volume-rendered image showing the patent graft and reconstructed celiac artery. 3D: 3-dimensional; CT: computed tomography; TEVAR: thoracic endovascular aortic repair

Preoperative computed tomography (CT) demonstrated occlusion at the origin of the Adamkiewicz artery and the presence of an extrathoracic collateral network. Thoracoabdominal aortic graft replacement (J Graft 28 mm; Japan Lifeline, Tokyo, Japan) was performed via an anterolateral thoracotomy with preservation of the latissimus dorsi muscle. The chest was entered through the left fifth intercostal space, and exposure was achieved using a rib cross thoracotomy with division of the sixth and seventh ribs.^6)^ The aneurysm was large and circumferentially adherent to the lung.

Partial extracorporeal circulation was established through right femoral arterial and right venous cannulation, and the aneurysm sac was opened. Aortic cross-clamping was intentionally avoided at this stage due to adhesions and the large size of the aneurysm to prevent injury. Dark red, non-thrombosed fluid was present within the aortic aneurysm, and marked backflow from the bronchial and intercostal arteries was observed. The intimal flap was excised to expose the pre-existing stent graft. After opening the aneurysm, the proximal stent graft was clamped and divided, and the proximal anastomosis was completed. For the distal anastomosis, residual aortic dissection within the false lumen precluded cross-clamping to avoid embolization and further intimal damage. Lower body circulatory arrest was performed for 8 min, with a lowest temperature of 33.1°C, and the distal stent graft was removed. Selective perfusion was provided to the celiac artery (CA) and the superior mesenteric artery (SMA). Thrombus within the false lumen was removed, and the thickened intima was excised. Distal anastomosis was created between the CA and SMA, followed by reconstruction of the CA (**Fig. 1D**). The extracorporeal circulation time was 134 min, and the total blood loss was approximately 3000 mL. Intraoperative transfusion included 4 units of red blood cells, 10 units of fresh frozen plasma (FFP), and 20 units of platelet concentrates. A decline in motor evoked potentials was observed after completion of the distal anastomosis.

On postoperative day 1, complete paraplegia was noted and diagnosed as SCI, and steroid pulse therapy (methylprednisolone 500 mg/day) with cerebrospinal fluid drainage was initiated immediately. Her blood pressure was well-controlled throughout the perioperative and postoperative periods. During the early postoperative period, unexplained thrombocytopenia and hemolytic anemia progressed. By postoperative day 2, PLT had declined to 48000/μL, with a hemoglobin level of 9.4 g/dL, an elevated lactate dehydrogenase (LDH) level of 1025 U/L, and a total bilirubin (T-Bil) of 19.3 mg/dL. AKI with an SCr level of 3.17 mg/dL developed, accompanied by fluid overload and anuria unresponsive to diuretics, necessitating the initiation of HD on postoperative day 3. PLT further dropped to 18000/μL on postoperative day 4. Hemolysis and hyperbilirubinemia continued to progress, with LDH and T-Bil levels reaching their maximums of 1361 U/L and 31.3 mg/dL, respectively, on postoperative day 6. In contrast, aspartate aminotransferase (AST, 80 U/L) and alanine aminotransferase (ALT, 15 U/L) remained near normal limits. The dissociation between elevated LDH and T-Bil and preserved transaminases made ischemic hepatic injury unlikely, and the clinical findings were not fully explained by DIC or organ ischemia alone. Hemolysis persisted, and the PLASMIC score was 7 on postoperative day 6, raising strong suspicion for TMA, including acquired TTP. Peripheral blood smears consistently showed schistocytes, and haptoglobin was reduced to <6 mg/dL. The direct Coombs test and HIT antibody were negative, excluding autoimmune hemolytic anemia and HIT.

As TMA with possible TTP could not be excluded, therapeutic PE using FFP was initiated on postoperative day 6. Metabolic alkalosis developed during daily PE with large volumes of plasma; therefore, from postoperative day 11, PE and HD were connected in a serial circuit, and treatment was continued with strict fluid and electrolyte management. Steroid therapy was changed to oral prednisolone 25 mg/day on postoperative day 6 and increased to 50 mg/day on postoperative day 12 to intensify immunosuppression against persistent hemolysis.

Subsequent testing demonstrated normal ADAMTS13 activity (>10%), excluding TTP, and the condition was diagnosed as postsurgical TMA triggered by aortic surgery within the spectrum of secondary TMA (**Table 1**). PE was performed daily until postoperative day 14. Urine output recovered around postoperative day 15, and by postoperative day 16, the PLT had risen to 103000/μL, with LDH and T-Bil trending toward normalization. Thereafter, PE was reduced to 3 sessions per week. After stabilization and recovery from the acute phase, the patient was transferred to a university hospital (Nagoya City University Hospital, Aichi, Japan) for further evaluation.

**Table 1:** Laboratory findings at the diagnosis of TMA

Diagnostic step	POD	Test (result)	Interpretation
Primary hepatic injury	1–6	AST/ALT (peak 80/11 U/L)	Excluded primary hepatocellular injury
		Total bilirubin (peak 31.3 mg/dL)	
Autoimmune hemolysis	2	Direct Coombs (negative)	Excluded AIHA (warm/cold)
		Cold agglutinin (1:4)	Alloantibodies from past transfusions
		Indirect Coombs (positive)	
Concurrent coagulopathy	2	ISTH overt DIC score (5 points)^†^	Overt DIC without coagulopathy
		HIT antibody IgG (negative)	Excluded HIT
MAHA confirmation	6	Peripheral blood smear (schistocytes: 5.0 per 100 WBCs)	Confirmed severe intravascular MAHA
		Haptoglobin (<6 mg/dL)	
TMA subtyping	6	PLASMIC score (7 points)	High risk; prompted empirical PE
	13*	ADAMTS13 activity (>10%)	TTP ruled out
	6	C3/C4/CH50 (78 mg/dL/14.2 mg/dL/27 U/mL)	Complement consumption
	6	ANA/ANCA (negative)	Excluded systemic autoimmune diseases

^†^The ISTH score of 5 consisted of platelets (2 pts), FDP/D-dimer (3 pts), PT-INR (0 pts), and fibrinogen (0 pts).

*ADAMTS13 testing was drawn on postoperative day 6, with the result reported on day 13.

ADAMTS13: a disintegrin and metalloproteinase with a thrombospondin type 1 motif, member 13; AIHA: autoimmune hemolytic anemia; ALT: alanine aminotransferase; ANA: antinuclear antibody; ANCA: antineutrophil cytoplasmic antibody; AST: aspartate aminotransferase; DIC: disseminated intravascular coagulation; FDP: fibrin/fibrinogen degradation products; HIT: heparin-induced thrombocytopenia; IgG: immunoglobulin G; ISTH: International Society on Thrombosis and Haemostasis; MAHA: microangiopathic hemolytic anemia; PE: plasma exchange; POD: postoperative day; PT-INR: prothrombin time-international normalized ratio; pts: points; TMA: thrombotic microangiopathy; TTP: thrombotic thrombocytopenic purpura; WBC: white blood cells

At the referral institution, her clinical condition remained stable; therefore, genetic testing was not performed. Her renal function gradually recovered, and she was successfully weaned off HD. Prednisolone was tapered and discontinued within a short period.

She was transferred back to the primary treating hospital (Nagoya City University East Medical Center, Aichi, Japan) on postoperative day 37 without requiring HD or steroid therapy. Her PLT had increased to the 200000/μL range, and her renal function had returned to the preoperative level (SCr 0.7–0.8 mg/dL). On postoperative day 77, she was transferred to a rehabilitation facility (**Fig. 2**). Over 3 years of follow-up, no recurrence of TMA has occurred without the use of complement inhibitors. Rehabilitation for her paraplegia resulted in a recovery of activities of daily living (ADL) sufficient for ambulation with a cane; although she developed a neurogenic bladder requiring clean intermittent catheterization, her bowel function remained controlled. Follow-up imaging has demonstrated no pseudoaneurysm or dilatation of the residual aorta.

**Fig. 2:**
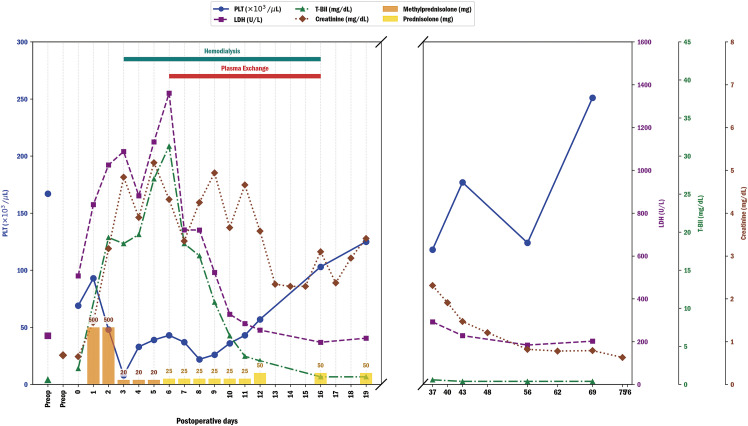
Overall in-hospital clinical course. Changes in key laboratory parameters and the treatment timeline, including preoperative baseline values, are presented. On postoperative day 11, a tandem circuit of PE and HD was initiated to manage severe electrolyte and acid–base disturbances while maintaining daily PE. HD: hemodialysis; LDH: lactate dehydrogenase; PE: plasma exchange; PLT: platelet count; Pre-op: preoperative baseline; T-Bil: total bilirubin

## Discussion

This case highlights that postsurgical TMA following thoracoabdominal aortic graft replacement can achieve sustained remission. In this case, prompt initiation of PE led to clinical stabilization despite TMA complicated by AKI and complete paraplegia, and remission has been maintained for 3 years without recurrence. Reports documenting long-term remission of this duration in postsurgical TMA remain limited.^3,4)^ These findings suggest that early recognition and timely intervention may permit durable remission.

Postsurgical TMA is usually caused by temporary triggers, such as surgical stress and endothelial damage.^4)^ However, previous reports on postsurgical TMA mostly consist of case reports and small series with short-term follow-up.^3,4)^ Although the exact mechanisms remain to be elucidated, we hypothesize that highly invasive aortic surgery inherently induced profound ischemia–reperfusion injury and a systemic inflammatory response syndrome.^3)^ This severe endothelial damage may have promoted the extensive release of unusually large von Willebrand factor (vWF) multimers, leading to a transient imbalance between ADAMTS13 and vWF.^1)^

The early onset of complete paraplegia in this case warrants careful consideration. As SCI is a recognized complication of this procedure, thorough preoperative planning has been undertaken. Reconstruction of the segmental arteries to maintain blood flow to the Adamkiewicz artery was deemed unnecessary, and a surgical strategy was implemented to preserve the extrathoracic collateral network; nevertheless, SCI developed. The concurrent TMA may have worsened the ischemic stress caused by the steal phenomenon from patent segmental arteries and circulatory arrest, contributing to the compromise of the extrathoracic collateral capacity.

In the acute management of severe TMA, international guidelines recommend the prompt initiation of empiric PE in combination with steroids while awaiting ADAMTS13 activity results.^5,7)^ In this case, steroid therapy had already been administered for SCI. Nevertheless, steroid therapy alone is insufficient to suppress the microvascular thrombus formation central to secondary TMA, including postsurgical TMA.^1,8)^ The favorable outcome observed in our patient was attributable to the early introduction of PE. This clinical course supports the notion that when a transient and potent trigger, such as surgery, is eliminated.

First, comprehensive screening for complement-related genetic mutations using next-generation sequencing and direct measurement of complement activation markers were not performed, leaving the complement overactivation as an indirect proof.^1,4)^ And, it is known that aortic aneurysms can induce consumptive coagulopathy accompanied by hyperfibrinolytic DIC.^9)^ Because its clinical picture overlaps with TMA, completely separating these 2 distinct pathologies in the acute phase remains inherently difficult.

## Conclusion

Thoracoabdominal aortic graft replacement can act as a potent trigger for postsurgical TMA, although underlying genetic predispositions were not evaluated. However, this case suggests that appropriate treatment may achieve favorable mid- to long-term outcomes without the need for maintenance therapy with complement inhibitors or steroids.

## References

[1] George JN, Nester CM. Syndromes of thrombotic microangiopathy. N Engl J Med 2014; 371: 654–66. 25119611 10.1056/NEJMra1312353

[2] Grigorescu M, Kamla CE, Wassilowsky D, et al. Severe acute kidney injury in cardiovascular surgery: thrombotic microangiopathy as a differential diagnosis to ischemia reperfusion injury. A retrospective study. J Clin Med 2020; 9: 2900. 32911781 10.3390/jcm9092900 PMC7565159

[3] Kamla CE, Grigorescu-Vlass M, Wassilowsky D, et al. Thrombotic microangiopathy following aortic surgery with hypothermic circulatory arrest: a single-centre experience of an underestimated cause of acute renal failure. Interact Cardiovasc Thorac Surg 2022; 34: 258–66. 34414411 10.1093/icvts/ivab231 PMC8929442

[4] van Herpt TTW, Timmermans S, van Mook W, et al. Postsurgical thrombotic microangiopathy and deregulated complement. J Clin Med 2022; 11: 2501. 35566627 10.3390/jcm11092501 PMC9100095

[5] Zheng XL, Vesely SK, Cataland SR, et al. ISTH guidelines for the diagnosis of thrombotic thrombocytopenic purpura. J Thromb Haemost 2020; 18: 2486–95. 32914582 10.1111/jth.15006 PMC8146131

[6] Tsukube T, Yoshimura M, Matsuda H, et al. Rib-cross thoracotomy for replacement of the thoracoabdominal or total descending aorta. J Vasc Surg 2003; 37: 219–21. 12514607 10.1067/mva.2003.49

[7] Scully M, Hunt BJ, Benjamin S, et al. Guidelines on the diagnosis and management of thrombotic thrombocytopenic purpura and other thrombotic microangiopathies. Br J Haematol 2012; 158: 323–35. 22624596 10.1111/j.1365-2141.2012.09167.x

[8] Legendre CM, Licht C, Muus P, et al. Terminal complement inhibitor eculizumab in atypical hemolytic–uremic syndrome. N Engl J Med 2013; 368: 2169–81. 23738544 10.1056/NEJMoa1208981

[9] Yamada S, Asakura H. Management of disseminated intravascular coagulation associated with aortic aneurysm and vascular malformations. Int J Hematol 2021; 113: 15–23. 33175341 10.1007/s12185-020-03028-z

